# Scalable preparation of osteogenic micro‐tissues derived from hESC‐derived immunity‐and‐matrix‐regulatory cells within porous microcarriers in suspension culture

**DOI:** 10.1111/cpr.13466

**Published:** 2023-05-17

**Authors:** Huike Ma, Tingting Gao, Liu Wang, Ali Mohsin, Jie Hao, Meijin Guo, Jun Wu

**Affiliations:** ^1^ State Key Laboratory of Bioreactor Engineering East China University of Science and Technology Shanghai China; ^2^ State Key Laboratory of Stem Cell and Reproductive Biology, National Stem Cell Resource Center Chinese Academy of Sciences Beijing China; ^3^ Institute for Stem Cell and Regeneration Chinese Academy of Sciences Beijing China; ^4^ State Key Laboratory of Stem Cell and Reproductive Biology Institute of Zoology, Chinese Academy of Sciences Beijing China

## Abstract

Bone defects (BDs), a prevalent clinically refractory orthopaedic disease, presently have no effective treatments. Mesenchymal stem cells (MSCs) can differentiate into osteoblasts and serve as potential seed cells for bone tissue engineering for BD treatment. However, the feasibility of using MSCs as seed cells for bone tissue engineering remains unclear. As a result, the critical issue of large‐scale cell‐scaffold preparation remains unresolved. In this study, we demonstrated for the first time that human embryonic stem cell‐derived MSCs, also known as immunity‐and‐matrix‐regulatory cells (IMRCs), could be inoculated into microcarriers to create osteogenic micro‐tissues appropriate for scalable production in 250 mL bioreactor. IMRCs were generally smaller than umbilical cord‐derived MSCs (UCMSCs) and could attach, migrate, proliferate and differentiate within the porous microcarriers, whereas UCMSCs could only attach to the surface of microcarriers. Osteogenic micro‐tissues generated from IMRCs‐seeded microcarriers significantly increased osteocalcin levels after 21 days of differentiation in a bioreactor. Furthermore, the expression levels of osteogenic biomarker genes/proteins such as alkaline phosphatase (ALP), osteocalcin (OCN), runt‐related transcription factor 2 (RUNX2), osteopontin (OPN) and osterix (OSX) were significantly higher than osteogenic micro‐tissues derived from UCMSCs‐seeded microcarriers. Our findings imply that IMRCs could potentially serve as seed cells for the scalable production of osteogenic micro‐tissues for BD treatment.

## Graphical abstract

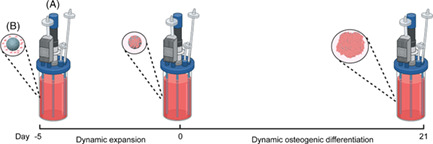

## 1 INTRODUCTION

Bone defect (BD) is a disease that impairs the structural integrity of bones due to congenital or acquired causes,^1^ such as acute bone loss, high‐energy trauma and infections caused by bone debridement or bone tumour resection.^2^ Without intervention, about 10% of BD progresses to nonunion.^3^ Annually, around 600,000 fractures with delayed or incomplete healing, occur in the United States, resulting in a cost of $200 billion.^4^ Currently, stem cell‐based bone tissue engineering (BTE) provide sgreat potential for BD healing. It aims to create biofunctional tissues that can integrate and degrade in vivo to fix tissue defects and replace part or all of the functions of lost or failing tissues and organs.^5^, ^6^

The selection of seed cells is one of the most significant components in BTE to treat BD. Mesenchymal stem cells (MSCs) are used as a cell population as a source of bone regeneration due to their excellent proliferation and osteogenic ability.^7^ However, donor circumstances, the origin of organs or tissues, and techniques of separation, purification and expansion all have a substantial impact on their quality.^8^ Human pluripotent stem cell (hPSC)‐derived MSCs, when compared to MSCs, provide an alternate source of MSCs due to their comparable or similar phenotypic, immunomodulatory and anti‐inflammatory properties.^9^ Furthermore, PSC‐derived MSCs have significant benefits in terms of differentiation efficiency, purity and cell quality consistency.^10^ A previous study reported that human embryonic stem cell (hESC)‐derived MSCs, also known as immune and stromal regulatory cells (IMRCs), are promising candidates for regenerative medicine due to their high proliferative capacity and lack of barriers to acquiring primary MSCs.^11^, ^12^, ^13^ The high‐purity MSCs can be derived from hESCs with the limited passage and good osteogenic ability.^14^ However, MSCs alone are insufficient for bone regeneration; generally, they are combined with scaffolds of appropriate shape, size and mechanical properties, in treatment.^15^, ^16^

Microcarriers are often considered suitable as spherical scaffolds for cell culture, growth and delivery.^17^ Porous microcarriers are widely used in tissue engineering due to their benefits in offering large surface area for cell growth, maintaining differentiated cell phenotypes and facilitating injection into target sites for repair or regeneration.^18^, ^19^ In addition, based on their interconnected and open pore structure, microcarriers provide a protective microenvironment for seed cell attachment, proliferation, migration, nutrient exchange and excretion of metabolic wastes.^20^, ^21^

Stem cell‐based BTE therapies require a minimum of 10^7^–10^9^ cells per treatment. To obtain the cell number needed for the clinical application, a stable and controlled expansion system is necessary for the large‐scale preparation of stem cells and their derived progenies.^22^ Suspension culture technology is a hopeful approach for large‐scale expansion and maintaining pluripotency of stem cells.^23^ Researchers previously injected human amniotic MSCs into CultiSpher‐S microcarriers and attempted osteogenic development in a spinner flask.^24^ Porous microcarriers in a suspension culture system were used to grow murine embryonic stem cell (mESC)‐derived osteoblasts and chondrocytes cells under feeder layer‐free and serum‐free conditions.^25^, ^26^ However, to date, there is a lack of research on whether hESC–MSCs can be expanded and differentiated into osteogenic cells in a bioreactor.

Therefore, the objectives of the study were to demonstrate the expansion and osteogenic differentiation ability of IMRCs in a bioreactor. It was demonstrated in this work for the first time that scaled‐up production of IMRCs was possible when combined with porous microcarriers in a bioreactor as well as directly differentiated to form osteogenic micro‐tissues.

## 2 MATERIALS AND METHODS

### 2.1 Cell monolayer culture in two‐dimensional condition

The hESC‐derived IMRCs and UCMSCs were obtained following a previous protocol.^13^ IMRCs and UCMSCs (passage 4, P4) were seeded on a 6‐well tissue culture plate at 1 × 10^4^ cells/cm^2^ and expanded in an atmosphere of 5.0% CO_2_ at 37°C using medium reported in the previous study.^13^ When cells were cultured to 70%–80% confluence, the medium was replaced with osteogenic medium, containing α‐MEM (Gibco, USA), 10% KOSR (Gibco, USA), 1.0% L‐glutamine (Gibco, USA), 100 nM dexamethasone (Sigma‐Aldrich, USA), 50 μg/mL L‐ascorbic acid (Sigma‐Aldrich, USA) and 10 mM β‐glycerophosphate (Sigma‐Aldrich, USA) for 21 days with changes of medium every 3 days. On Day 21, quantification of Alizarin red S (ARS) staining was performed by a colorimetric quantitative detection kit (Genmed, USA).

### 2.2 Cell expansion in a bioreactor

3D TableTrix™ (CytoNiche, China) are macroporous microcarriers with a diameter of 100–200 μm.^27^ The dry microcarriers (200 mg) were rehydrated in the medium at 37°C until next step. IMRCs and UCMSCs (P4) were seeded onto microcarriers in growth media at densities of 0.5 × 10^5^, 1.0 × 10^5^, 1.5 × 10^5^ and 2.0 × 10^5^ cells/mL, respectively, in a 250 mL siliconized bioreactor (Eppendorf, USA) with continuous stirring. Following the cell seeding procedure, the bioreactor culture settings (150–210 rpm) were set to prevent microcarriers from settling to the bottom of bioreactor, pH was set to 7.2, temperature was set at 37°C and dissolved oxygen (DO) tension was set to 40%. The culture was maintained for up to 7 days, and the culture medium was replenished with fresh medium every day, glucose was added daily based on cell density.

### 2.3 Osteogenic differentiation in a bioreactor

**FIGURE 1:**
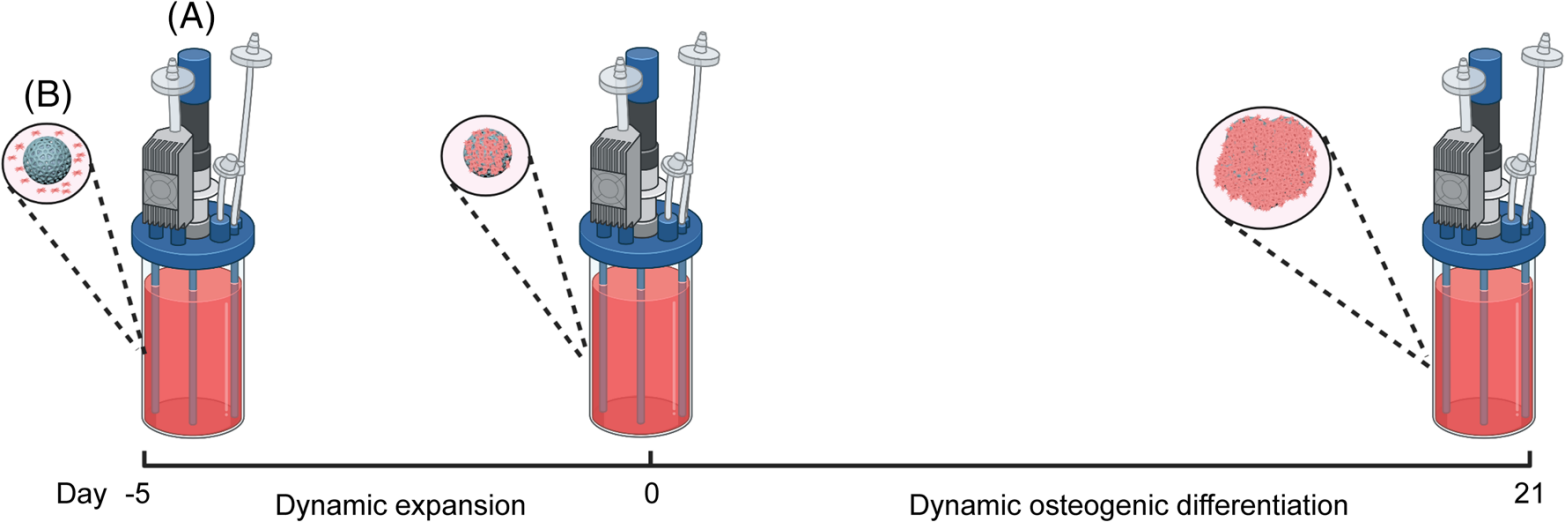
Schematic diagram of osteogenic differentiation in a bioreactor (A) DASbox mini bioreactor (B) 3D TableTrix microcarriers.

IMRCs and UCMSCs (P4) were seeded onto microcarriers in growth media at densities of 1.5 × 10^5^ and 1.0 × 10^5^ cells/mL, respectively. The cell‐seeded microcarriers were cultured in suspension in the growth medium for 5 days before being replaced with the osteogenic differentiation medium for 21 days cultured in suspension in a bioreactor. The osteogenic differentiation medium was refreshed after every 3 days. The schematic diagram is illustrated in Figure 1.

### 2.4 Cell counting and metabolism analysis

Every 24 h, 1 mL suspension of cell‐laden microcarriers in a bioreactor was centrifuged at 1500 rpm for 3 min, and the supernatant was removed carefully. Then, 1 mL 3D FloTrix Digest (CytoNiche, China) was added and incubated at 37°C for 30 min. Cell numbers were counted by the AOPI dual‐fluorescence assay using an automatic cell counter according to its user manual (CountStar Rigel S2, Ruiyu BioTech, China).

Cell supernatant was collected from the bioreactor immediately prior to medium addition or exchange. The concentrations of glucose and lactate were detected with the SBA‐Biosensing Analyser (Yanhe Biological, China).

### 2.5 Live/dead staining of cells on microcarriers

Cell viability on microcarriers was evaluated on Days 1, 3, 5 and 7. In brief, a 200 μL solution of cell‐laden microcarriers was rinsed in PBS and allowed to settle at the bottom of the wells microplate. They were stained with calcein AM and propidium iodide for 15 min, then rinsed with PBS and photographed using an inverted fluorescence microscope (Zeiss, Germany). Red fluorescence indicates dead cells, whereas green fluorescence indicates live ones.

### 2.6 Scanning electron microscopy and energy dispersive spectrometer

Cell‐laden microcarriers were collected on Days 1, 3, 5 and 7 of expansion phase and Day 21 of osteogenic differentiation phase in a bioreactor. The samples were washed three times with PBS, fixed in 2.5% glutaraldehyde at 4°C overnight, dehydrated in ascending grades of ethanol and air‐dried, and examined under scanning electron microscopy (SEM) (Hitachi, Japan). In addition, on Day 21, the cell‐laden microcarriers were collected for energy dispersive spectrometer (EDS) analysis (Japan).

### 2.7 Cell distribution on microcarriers

Cell‐laden microcarriers were taken on Day 4 from a bioreactor and fixed in 4.0% paraformaldehyde at 4°C overnight before being permeabilized with 0.1% Triton X‐100 for 10 min. The cells were then stained with phalloidin (Invitrogen, USA) and Hoechst 33342 (Invitrogen, USA). Next, they were cut into 30 μm sections, finally, the distribution of cells within the microcarrier was observed using confocal laser scanning microscopy (Zeiss, Germany).

### 2.8 Real‐time fluorescence quantitative PCR

Cells were harvested on Day 21 of osteogenic differentiation, and the RNA extraction of harvested cells was performed according to the manufacturer's instructions. Briefly, 2 μg of mRNA was reverse transcribed into cDNA using a PrimeScript™ First‐Strand cDNA Synthesis Kit (TaKaRa, Japan). Genes expression of alkaline phosphatase (ALP), osteocalcin (OCN) and runt‐related transcription factor 2 (RUNX2) were analysed. qPCR was performed and analysed on LightCycler 480 (Roche Applied Science, USA). GAPDH was used as a housekeeping gene. Primer sequences are listed in Table S1.

### 2.9 Transcriptome analysis

The RNA of cells harvested on Day 21 of osteogenic differentiation in 2D was sent to Annoroad Company for mRNA sequencing (RNA‐Seq). Differential genes in the samples were analysed by gene ontology (GO) enrichment analysis.

### 2.10 Immunocytochemistry

Cell‐laden microcarriers were harvested on Day 21 of osteogenic differentiation in a bioreactor, fixed overnight in 4.0% paraformaldehyde, cut into 8 μm thick sections and permeabilized with 0.3% Triton X‐100 for 10 min and blocked with 2% bovine serum album (BSA) for 60 min at room temperature. Then, the sample sections were stained with 10 μg/mL mouse anti‐osteocalcin antibody (R&D systems, USA) overnight at 4°C. The aggregates sections were washed three times with PBS buffer and then incubated with 1:200 Fluorescein (Cy3) donkey anti‐mouse IgG (H + L) (Jackson ImmunoResearch, USA) secondary antibodies in 2.0% BSA for 60 min at room temperature in the dark. Next, the nuclei were stained with Hoechst 33342 and were imaged under confocal laser scanning microscopy (Zeiss, Germany).

### 2.11 Western blot

RIPA Lysis Buffer (CWbio, China) containing protease inhibitors was used to collect samples (Roche, USA). Twenty grams of proteins were separated using 4%–20% gradient SDS‐PAGE gels (GenScript, USA) and transferred to PVDF membranes (Millipore, Germany). The membranes were blocked for 1 h at room temperature with 1% BSA before being incubated overnight at 4°C with primary antibodies: mouse anti‐RUNX2 antibody (Abcam, UK) at 1:1000, rabbit anti‐osteopontin (OPN) antibody (Affinity, USA) at 1:1000 and mouse anti‐GAPDH antibody (Proteintech, USA) at 1:10,000. Then, the membranes were washed with TBS and with Tween‐20 (TBST) three times, and incubated for 1 h with a secondary antibody: anti‐mouse IgG antibody (HRD) (Sigma‐Aldrich, USA) and anti‐rabbit IgG antibody (HRD) (Sigma‐Aldrich, USA) at room temperature. Images were obtained using the ChemiDoc XRS + imaging system (Bio‐Rad, USA).

### 2.12 Statistical analysis

Numerical data were represented as mean ± standard deviation. The difference between groups was evaluated by Student's *t*‐test. Statistical significance was *p < 0.05, **p < 0.01 and ***p < 0.001.

## 3 RESULTS

### 3.1 IMRCs exhibited superior osteogenic potential when grown under 2D condition

**FIGURE 2:**
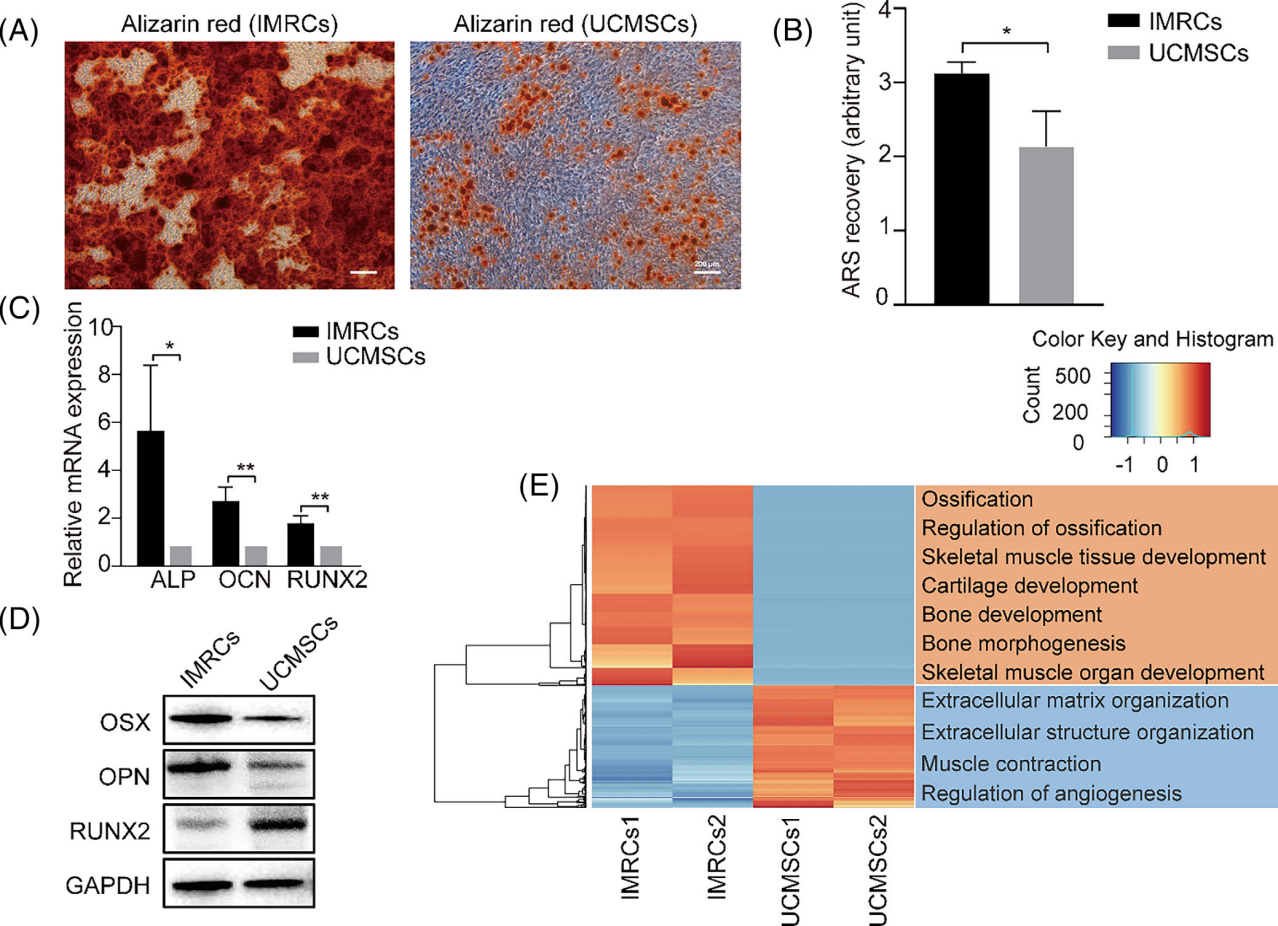
Osteogenic ability of IMRCs and UCMSCs grown under 2D condition. (A) Representative images of ARS staining. (B) ARS quantification assay for mineralization. (C) The osteogenic gene expression levels of ALP, OCN and RUNX2 by qPCR. (D) The osteogenic protein levels of OSX, OPN and RUNX2 by WB. (E) The differentially expressed genes between IMRCs and UCMSCs.

IMRCs and UCMSCs at P4 were cultivated for 21 days to determine their osteogenic potential. As shown in Figure 2, cells produced mineralizing ARS‐stained monolayers, and larger nodules were seen on IMRC monolayers (Figure 2A). Furthermore, results showed that IMRCs were superior to UCMSCs in terms of osteogenic potential based on the quantity of ARS‐stained minerals (Figure 2B). qPCR analysis showed that the mRNA expression levels of osteoblast markers showed higher expression in IMRCs than UCMSCs such as ALP (5.67‐fold), OCN (2.86‐fold) and RUNX2 (1.87‐fold) (Figure 2C). Meanwhile, WB analysis indicated that OSX and OPN were higher expressed in IMRCs, whereas RUNX2 was down‐regulated (Figure 2D). RUNX2 is a transcription factor involved in osteogenic differentiation. It was weakly expressed in undifferentiated MSCs, up‐regulated in osteogenic precursor cells, highest in immature osteoblasts and down‐regulated in mature osteoblasts.^28^, ^29^ So, IMRCs formed mature osteoblasts after osteogenic differentiation. Differential gene expression analysis of IMRCs and UCMSCs showed up‐regulation of genes in osteogenesis, bone morphogenesis, bone development pathway and several bone‐related pathways in IMRCs (Figure 2E). Collectively, these results demonstrated that IMRCs exhibited superior osteogenic properties.

### 3.2 IMRCs possessed faster expansion capability when grown in a 3D culture

**FIGURE 3:**
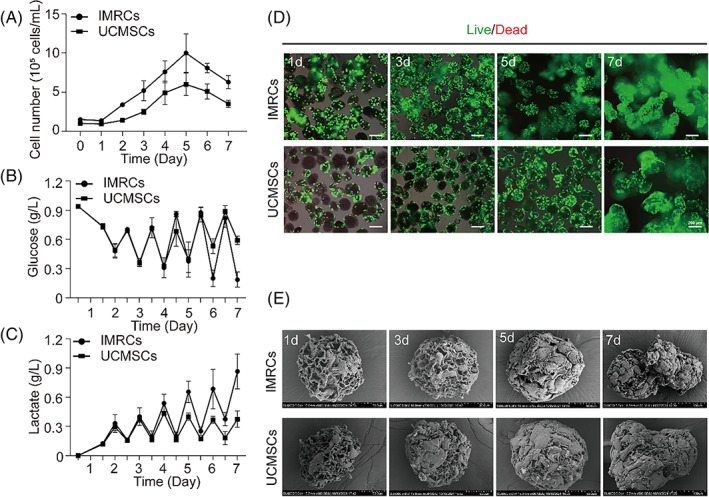
Expansion ability of IMRCs and UCMSCs grown in the bioreactor. (A) The growth curves of IMRCs and UCMSCs. (B) Residual glucose concentration. (C) Lactic acid concentration. (D) Live (green)–dead (red) staining of cells. (E) SEM images of cells on porous microcarriers.

Porous microcarriers were utilized to expand MSCs grown under 3D conditions in a bioreactor, and it was found that seeding density affects cell proliferation rate.^27^ To explore the optimal seeding density for expansion to increase the utilization of microcarriers, DASbox was seeded at cell densities of 0.5 × 10^5^, 1.0 × 10^5^, 1.5 × 10^5^ and 2.0 × 10^5^ cells/mL with TableTrix for 7 days in a bioreactor. The optimal inoculation densities of IMRCs and UCMSCs were 1.5 × 10^5^ and 1 × 10^5^ cells/mL, respectively (Figure S1). When cells were grown in suspension in a bioreactor, the growth curves showed a typical S type in the expansion phase, and the maximum cell density of IMRCs and UCMSCs were 9.99 × 10^5^ and 5.99 × 10^5^ cells/mL, respectively (Figure 3A), indicating that the higher density of IMRCs can be achieved than UCMSCs when they were cultured in bioreactors with optimal seed densities. As a result, as compared to UCMSCs culture, the residual glucose concentrations for IMRCs with greater glucose consumption rates over the whole suspension process were slightly lower (Figure 3B), and the accumulated concentration of lactic acid was higher for IMRCs (Figure 3C). However, the maximum lactic acid concentration was 0.86 g/L (9.55 mM) for IMRCs and 0.44 g/L (4.88 mM) for UCMSCs, which are both lower than the minimum toxic concentration of lactic acid to mammalian cells of 24.8 mmol/L. Furthermore, the survival of cells grown on the porous microcarriers was detected by live‐dead cell staining. Fluorescence staining of cells confirmed that almost all cells were alive on microcarriers throughout the entire culture process (Figure 3D). SEM was used to observe the surface characteristics of cell‐laden porous microcarriers. On Day 1, IMRCs and UCMSCs were attached to single porous microcarriers, and by Day 5, the individual porous microcarriers were nearly full of cells and began to aggregate. On Day 7, it was observed that cells produced dense aggregates with uneven aggregate surfaces (Figure 3E). Conclusively, a large number of IMRCs with high cell viability could be generated in a bioreactor culture system with porous microcarriers.

### 3.3 Distribution of IMRCs in cell‐loaded porous microcarriers

**FIGURE 4:**
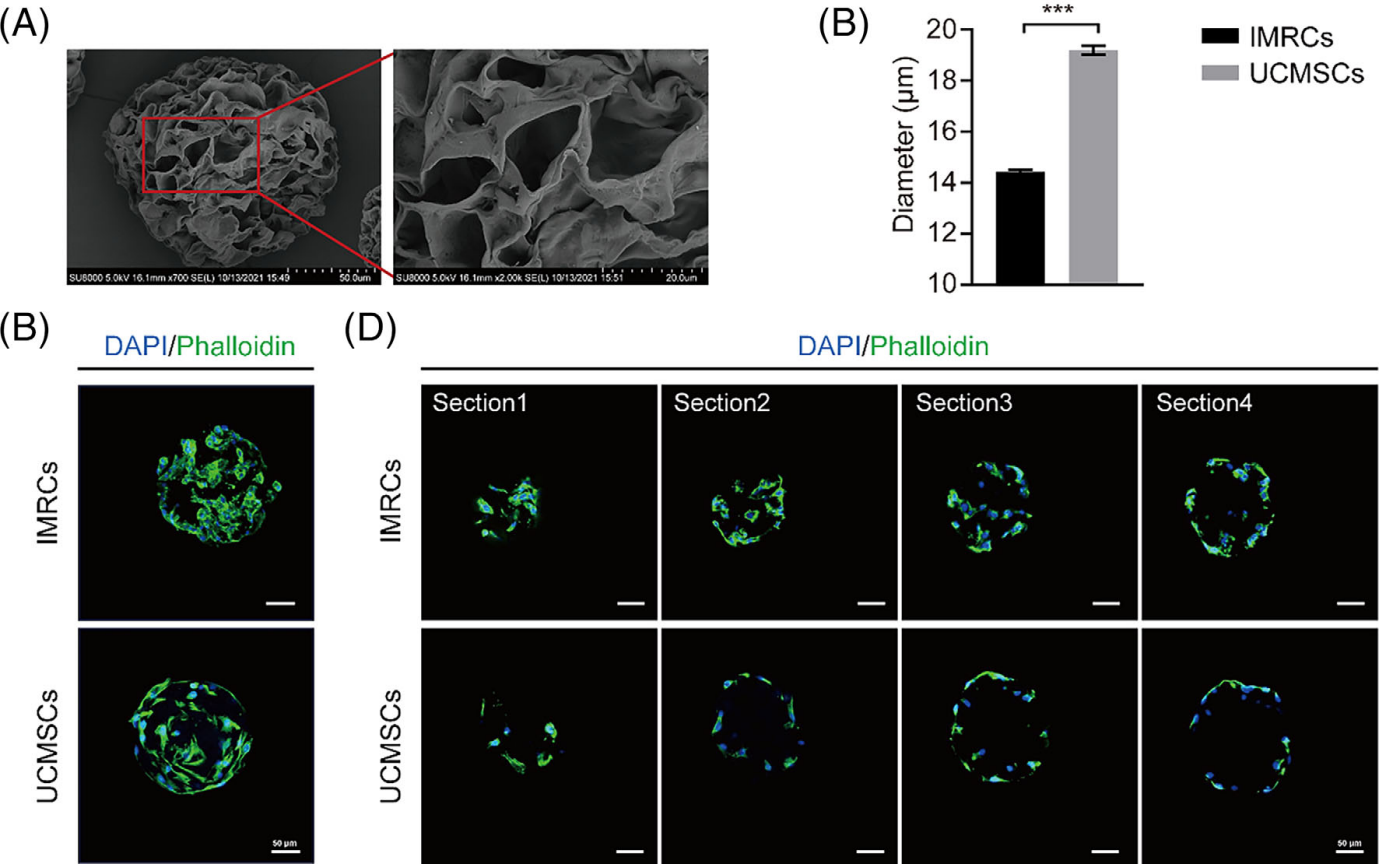
The distribution of IMRCs and UCMSCs on porous microcarriers. (A) SEM images of porous microcarriers. (B) The diameters of cells on microcarriers. (C) Distribution of cells on the surface of the porous microcarriers. (D) Distribution of cells on the inside of the porous microcarriers.

Porous scaffolds feature a high density of interconnected 3D porous structures, making them more conducive to cell migration and nutrient supply than hydrogels.^30^, ^31^ The average inner pore size of TableTrix porous microcarriers was 20 ± 5.7 μm.^27^ SEM (Figure 4A) observation of the hydration of TableTrix microcarriers revealed that the diameters of IMRCs and UCMSCs were 14 ± 0.36 μm and 19 ± 0.70 μm, respectively (Figure 4B) being smaller than the pores of microcarriers. In principle, they can grow in microcarrier holes. Immunofluorescence staining of cells was observed using confocal microscopy and revealed that IMRCs and UCMSCs were distributed on the surface of the porous microcarriers (Figure 4C). However, the section staining results showed that IMRCs were distributed in the centre and at the edges of the microcarriers, while UCMSCs were just distributed at the edges (Figure 4D). Therefore, the results presented here demonstrated that only IMRCs can grow and migrate in and out of porous microcarriers in a bioreactor.

### 3.4 Superior osteogenic properties of osteo‐microtissues derived from IMRCs

**FIGURE 5:**
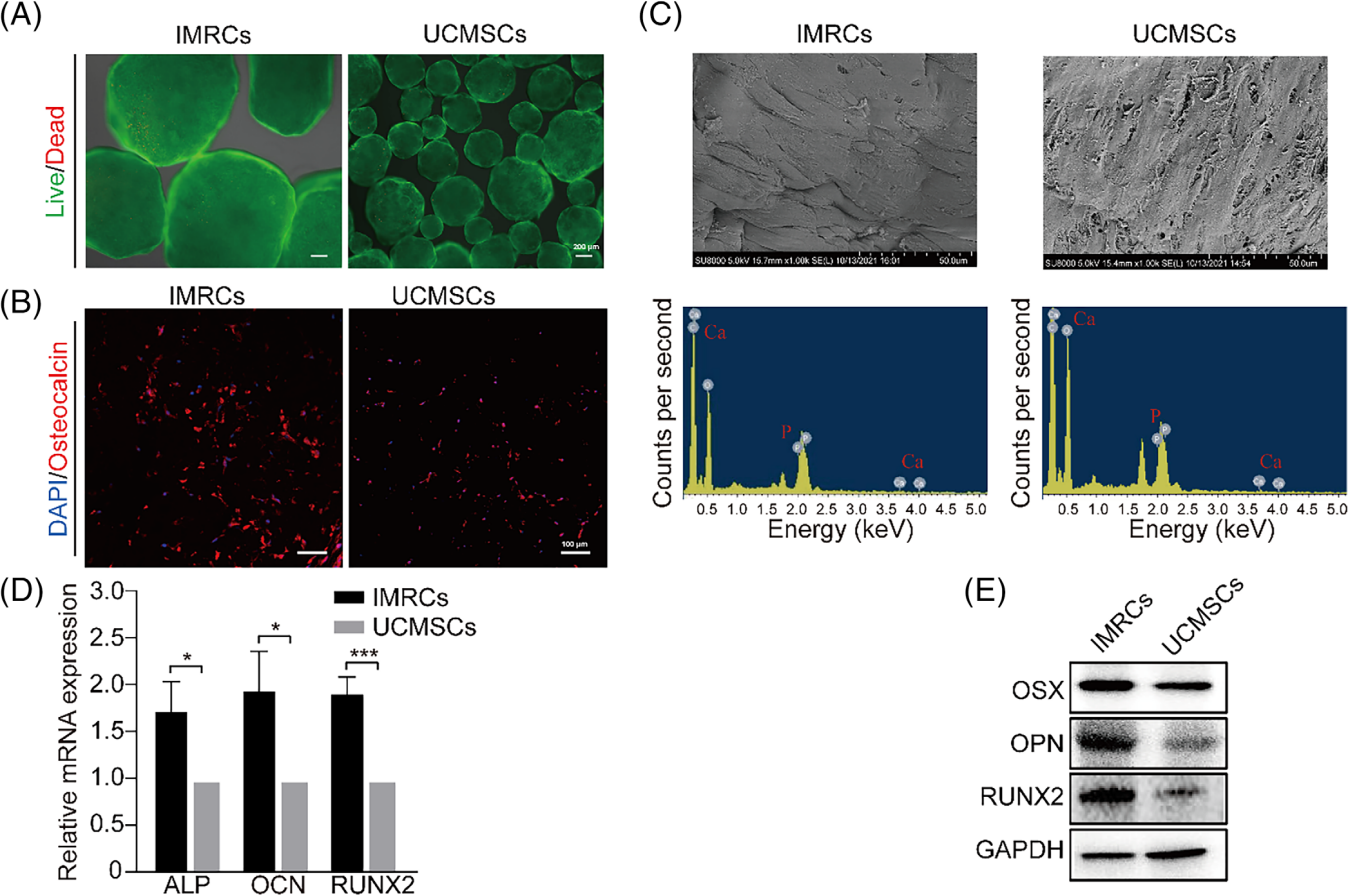
Osteogenic ability analysis of IMRCs and UCMSCs in a bioreactor. (A) Live (green)–dead (red) staining of cells. (B) Osteocalcin was probed with anti‐osteocalcin (red) and stained for nuclei with Hoechst 33342 (blue). (C) SEM images and EDS spectrogram. (D) The expression of osteogenic‐related genes. (E) The expression of osteogenic‐related proteins.

The cell‐loaded porous microcarriers underwent dynamic osteogenic differentiation in the bioreactor for 21 days. The size of aggregates formed by IMRCs and UCMSCs were 1442 ± 298 μm and 575 ± 238 μm, respectively. Live‐dead staining showed red fluorescence in all micro‐structures, indicating the presence of dead cells in the aggregates (Figure 5A). Immunocytochemistry was performed to indicate that the cells had differentiated into osteoblasts, as evidenced by the presence of osteocalcin in cells (Figure 5B). In SEM micrographs, IMRCs appeared more compact on the microcarriers. EDS analysis of IMRCs and UCMSCs aggregates further confirmed the presence of calcium and phosphorous (Figure 5C). To compare the osteogenic potential of osteo‐microtissues from IMRCs and UCMSCs, osteogenic marker genes were detected. Interestingly, the expression levels of ALP (1.72‐fold), OCN (1.94‐fold) and RUNX2 (1.91‐fold) in IMRCs were higher than UCMSCs (Figure 5D). Furthermore, WB analysis also confirmed the increased expression of RUNX2, OPN and OSX proteins expression in osteo‐microtissues from IMRCs (Figure 5E). Therefore, these results demonstrated that cells were seeded onto microcarriers to form osteo‐microtissue after osteogenic differentiation. Moreover, the osteogenic capacity of IMRCs‐seeded microcarriers formed osteogenic micro‐tissues was superior to the osteogenic microtissues formed from UCMSC‐seeded microcarriers.

## 4 DISCUSSION

MSCs have a wide range of application prospects in tissue engineering. Like MSCs, IMRCs have immune privileges that allow them to evade allogeneic immune responses. In our previous study, we demonstrated the potential of IMRCs, in which a soft agar assay was performed, and no colonies were formed.^13^ In addition, IMRCs could not form any tumour in immunodeficient mice after injection in vivo.^13^ So, it is concluded that the IMRCs have promising application prospects in BTE. Moreover, it is well known that the expansion and osteogenic abilities of stem cells are crucial for the application of BTE,^32^ and our comparative study based on two factors of IMRCs and UCMSCs demonstrated this. In the expansion stage, the number of IMRCs was higher than UCMSCs in a bioreactor and only IMRCs could migrate to the pores of porous microcarriers for cell expansion. In the stage of osteogenic differentiation, a large number of osteogenic micro‐tissues were formed based on the large‐scale expansion in a bioreactor. Furthermore, when compared to UCMSCs, IMRCs showed strong expression in osteoblast‐specific genes and proteins following osteogenic differentiation, suggesting that IMRCs have good osteogenic characteristics in 3D settings.

Goh et al. used Cytodex 3 microcarrier‐expanded human foetal MSC (hfMSC) cultures to achieve the density of 8 × 10^5^ cells/mL.^33^ Our current study revealed that while the density of 9.99 × 10^5^ cells/mL of IMRCs was achieved in a bioreactor, 15 T‐75 flasks of IMRCs cultivated in monolayer were required to yield the same quantity. Thus, the 3D system may be readily used for large‐scale production of IMRCs at a low cost. More intriguingly, PSC‐derived MSCs are morphologically more smaller in size than BM‐MSCs.^34^ In this study, the area utilization rate of microcarriers was further enhanced because the diameter of IMRCs was lower than that of UCMSCs. It can easily be scaled up to produce as many cells as necessary for transplantation.

To treat bone injury, osteogenic micro‐tissues should be produced on a large scale. Only a few researchers have explored the use of bioreactors in the tissue engineering of bone constructs from MSCs. Previous studies found that MSCs can combine CMPs particles to form osteogenic micro‐tissues in spinner flask.^21^, ^35^ However, this was the first time IMRCs were differentiated to osteoblasts in a bioreactor using TableTrix microcarriers. It might be used as cell‐scaffold composites for possible BD treatment.

However, there were some limitations in this study. For example, the presence of dead cells in osteogenic micro‐tissues needs to be further investigated and a detection method to quantify the amount of dead cells in the osteogenic micro‐tissues is required. Moreover, the exact mechanism underlying this discrepancy between the osteogenic potential of IMRCs and UCMSCs is currently unknown. It is possible that several factors, such as culture conditions (including oxygen tension and mechanical stimulation), microcarrier materials and reaction systems, may affect cell proliferation and differentiation.^36^, ^37^ Therefore, further studies are required to optimize and explore the culture parameters in a bioreactor.

## 5 CONCLUSION

To summarize, we investigated the proliferative and osteogenic potential of IMRCs and UCMSCs. The results showed that IMRCs had a greater proliferative and osteogenic capacity between 2D and 3D cultures. Additionally, the porous microcarriers were adequate for promoting the proliferation and osteogenic differentiation of IMRCs compared with UCMSCs, which can differentiate into osteogenic micro‐tissues, allowing for the manufacture of millimetre‐sized osteogenic micro‐structures in a bioreactor. In conclusion, these findings suggest that IMRCs might be employed as seed cells in BTE.

## AUTHOR CONTRIBUTIONS

Huike Ma and Jun Wu designed the work. Huike Ma performed the experiments and the data analysis. Huike Ma, Jun Wu, Ali Mohsin and Meijin Guo wrote the manuscript. Tingting Gao performed a few experiments and the data analysis. The manuscript language was revised by Ali Mohsin. Jie Hao and Liu Wang contributed essential reagents or tools. All authors read and approved the final manuscript.

## FUNDING INFORMATION

This research was supported by the Strategic Priority Research Program of the Chinese Academy of Sciences (No. XDA16030702). The authors thank the National Stem Cell Resource Center, Chinese Academy of Sciences for its support.

## CONFLICT OF INTEREST STATEMENT

The authors declare no conflicts of interest.

## Supporting information


**Figure S1.** Effect of different inoculum densities on the expansion and metabolism of IMRCs and UCMSCs. (a) The growth curves of IMRCs and UCMSCs under different inoculation densities. (b) Residual glucose concentration. (c) Lactic acid concentration.

**Table S1.** List of primers used in this study.

## Data Availability

The datasets generated during and/or analysed during the current study are available from the corresponding author on reasonable request.
