# Supplementary material for: Scalable preparation of osteogenic micro‐tissues derived from hESC‐derived immunity‐and‐matrix‐regulatory cells within porous microcarriers in suspension culture
Source: Cell Prolif. 2023 May 17;56(5):e13466. doi: 10.1111/cpr.13466 (PMC10212705; doi:10.1111/cpr.13466)
Supplement: Supplementary file 1 — Figure S1. Effect of different inoculum densities on the expansion and metabolism of IMRCs and UCMSCs. (a) The growth curves of IMRCs and UCMSCs under different inoculation densities. (b) Residual glucose concentration. (c) Lactic acid concentration. [file CPR-56-e13466-s002.docx]

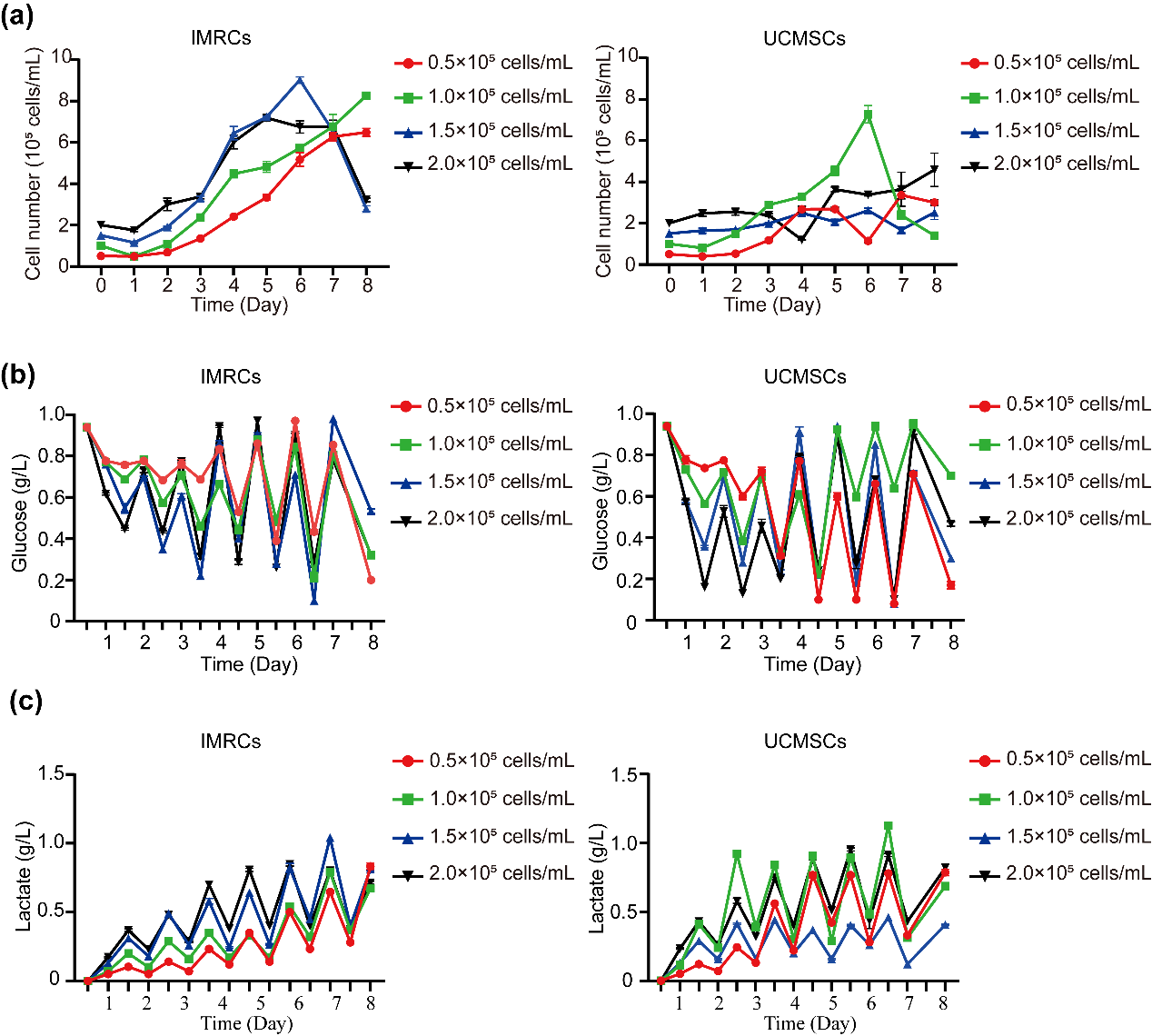


**Figure S1.** Effect of different inoculum densities on the expansion and metabolism of IMRCs and UCMSCs. (a) The growth curves of IMRCs and UCMSCs under different inoculation densities. (b) Residual glucose concentration. (c) Lactic acid concentration.
