# Supplementary material for: Scalable preparation of osteogenic micro‐tissues derived from hESC‐derived immunity‐and‐matrix‐regulatory cells within porous microcarriers in suspension culture
Source: Cell Prolif. 2023 May 17;56(5):e13466. doi: 10.1111/cpr.13466 (PMC10212705; doi:10.1111/cpr.13466)
Supplement: Supplementary file 2 — Table S1. List of primers used in this study. [file CPR-56-e13466-s001.docx]

**Supplementary information**

**Table S1.** List of primers used in this study.

| **primers** | **primer sequences** |
| --- | --- |
| GAPDH-F | CTCTGCTCCTCCTGTTCGAC |
| GAPDH-R | CGACCAAATCCGTTGACTCC |
| ALP-F | ACTGGTACTCAGACAACGAGAT |
| ALP-R | ACGTCAATGTCCCTGATGTTATG |
| OCN-F | CACTCCTCGCCCTATTGGC |
| OCN--R | CCCTCCTGCTTGGACACAAAG |
| RUNX2-F | CCGCCTCAGTGATTTAGGGC |
| RUNX2-R | GGGTCTGTAATCTGACTCTGTCC |
